# Connection control implications in a distributed plasticity cerebellar model

**DOI:** 10.1186/1471-2202-14-S1-P329

**Published:** 2013-07-08

**Authors:** Niceto R Luque, Jesús A Garrido, Richard R Carrillo, Eduardo Ros

**Affiliations:** 1 Dept. of Computer Architecture and Technology. University of Granada, Granada, Spain; 2 Dept. of Brain and Behavioral Sciences. University of Pavia, Consorzio Interuniversitario per le Scienze Fisiche della Materia (CNISM), Pavia, Italy

## 

The cerebellum is one of the parts of the brain which has aroused more curiosity amongst neuroscientists. Indeed, several forms of plasticity mechanisms have been reported at several sites of the cerebellar model circuitry, thus including plasticity mechanisms not just at parallel fibers (a well-accepted plasticity site) but also at synaptic inputs of deep cerebellar nuclei (DCN) (from mossy fibers (MFs) [1,2]and Purkinje cells (PCs) [3,4]). The Marr and Albus model already hypothesized that parallel fiber (PFs) →Purkinje cell synapses presented both long-term potentiation (LTP) [5,6] and long-term depression (LTD) [5-7] plasticity so as to correlate the activity at parallel fibers with the incoming error signal through climbing fibers. Nevertheless, in subsequent studies, it has been demonstrated that many sites in the cerebellum show traces of plasticity [8-10]. But the way in which those distributed plasticity mechanisms may improve the operational capabilities of the cerebellum is still an open issue.

In this work we propose that the synaptic plasticity of mossy-fiber-to-deep-cerebellar-nucleus-cell and Purkinje-cell-to-deep-cerebellar-nucleus-cell may regulate the effect of Purkinje-cell activity on the cerebellar output, behaving as a distributed homeostatic mechanism [11]. The plasticity at the DCN afferents helps to keep the Purkinje-cell activity in an adequate range independently of the magnitude required for the cerebellar output, thus improving the precision of this output signal.

Since these plasticity mechanisms are capable of adapting the cerebellar behavior in the long-term, it is of necessity the presence of fast feedback for motor activities in order to perform precise movements. Thus, the presented work also explores the control implication that the inferior-olive-to-deep-cerebellar-nucleus-cell connection may possess in conjunction with the previously suggested plasticity mechanisms. As it is widely assumed, the climbing fiber activity that our cerebellar model implements is considered to be a teaching signal (targeting Purkinje cells). But we also explore its potential role as a control signal over the cerebellar output (targeting the deep cerebellar nuclei).

To investigate all these proposals, we have embedded the cerebellar model in a feed-forward control loop which is connected to a simulated 3-degree-of-freedom robot model.
